# Detection of Threshold-Level Stimuli Modulated by Temporal Predictions of the Cerebellum

**DOI:** 10.1523/ENEURO.0070-24.2024

**Published:** 2024-04-23

**Authors:** Lau M. Andersen, Sarang S. Dalal

**Affiliations:** ^1^Center of Functionally Integrative Neuroscience (CFIN), Aarhus University, Aarhus C 8000, Denmark; ^2^Aarhus Institute of Advanced Studies (AIAS), Aarhus University, Aarhus C 8000, Denmark; ^3^Department for Linguistics, Cognitive Science and Semiotics, Aarhus University, Aarhus C 8000, Denmark

**Keywords:** action, cerebellum, expectation, magnetoencephalography, prediction, thalamus

## Abstract

The cerebellum has the reputation of being a primitive part of the brain that mostly is involved in motor coordination and motor control. Older lesion studies and more recent electrophysiological studies have, however, indicated that it is involved in temporal perception and temporal expectation building. An outstanding question is whether this temporal expectation building cerebellar activity has functional relevance. In this study, we collected magnetoencephalographic data from 30 healthy participants performing a detection task on at-threshold stimulation that was presented at the end of a sequence of temporally regular or irregular above-threshold stimulation. We found that behavioral detection rates depended on the degree of irregularity in the sequence preceding it. We also found cerebellar responses evoked by above-threshold and at-threshold stimulation. The evoked responses to at-threshold stimulation differed significantly, depending on whether it was preceded by a regular or an irregular sequence. Finally, we found that detection performance across participants correlated significantly with the differences in cerebellar evoked responses to the at-threshold stimulation, demonstrating the functional relevance of cerebellar activity in sensory expectation building. We furthermore found evidence of thalamic involvement, as indicated by responses in the beta band (14–30 Hz) and by significant modulations of cerebello-thalamic connectivity by the regularity of the sequence and the kind of stimulation terminating the sequence. These results provide evidence that the temporal expectation building mechanism of the cerebellum, what we and others have called an internal clock, shows functional relevance by regulating behavior and performance in sensory action that requires acting and integrating evidence over precise timescales.

## Significance Statement

We demonstrate that the predictions that the cerebellum builds are functionally relevant for sensory detection, showing that participant performance on threshold-level stimuli detection correlates with cerebellar activation. We find that cerebellar activation and behavioral performance depend on the temporal predictability of when the target stimulus will appear. We furthermore find suggestive evidence of a thalamic contribution translating the cerebellar prediction into informed behavior, as we not only find evidence of a thalamic activation, but also of cerebello-thalamic functional connectivity that is modulated differently according to whether threshold-level stimuli were perceived by participants. Overall this supports the idea of the cerebellum as having predictive temporal functions and intriguingly provides evidence of the mechanism that translates cerebellar predictions into informed behavior.

## Introduction

The cerebellum is being studied with renewed interest after it has become clear that its function is not only one of motor coordination but also activity related to cognition (King et al., 2019; Andersen et al., 2020). Its cognitive abilities have been thought to include detection of stimulus rhythmicity (Ivry, 1997). In humans, evidence for this ability has come from lesion studies of cerebellar patients (Ivry, 1993). Recent studies, however, have provided electrophysiological, noninvasive evidence of cerebellar involvement in temporal sensory prediction (Herrojo Ruiz et al., 2017; Andersen and Lundqvist, 2019; Andersen and Dalal, 2021). The confidence in these findings has been further supported by a recent study demonstrating that both electroencephalography (EEG) and magnetoencephalography (MEG) are sensitive to cerebellar activations (Samuelsson et al., 2020). These studies show that cerebellar activity correlates with temporal predictability. However, the functional relevance of the cerebellum in potentially guiding behavior remains unclear. Given such an influence on behavior, it is natural to ask what neural connections would make it possible to elicit informed motor action. Connections from the cerebellum to the primary motor cortex would indicate that such an influence is possible. Such a connection has been reported in monkeys to take place through the thalamus (Orioli and Strick, 1989; Bostan et al., 2013). Furthermore, evidence of functional connectivity between the cerebellum, thalamus, and the primary motor cortex has been reported in natural tremor and essential tremor using MEG (Gross et al., 2002; Pollok et al., 2008; Schnitzler et al., 2009). Thus, it is possible that the thalamus is instrumental in making sensory expectations in the cerebellum informative to behavior due to the cerebellum's connections through the thalamus to the primary motor cortex. Besides anatomical connections to the thalamus, anatomical connections have also been reported between the cerebellum, the thalamus and the basal ganglia (Bostan and Strick, 2010; Bostan et al., 2013). This is interesting because basal ganglia are thought to be involved in decision-making (Bogacz and Gurney, 2007; Ding and Gold, 2013). Interestingly, we earlier found evidence of thalamic responses [Andersen and Dalal (2022), their Supplementary Material 3: New Figure 3] in the beta band (14–30 Hz). This makes it possible to test whether the cerebellum and the thalamus show functional connectivity, if we replicate the finding of thalamic activity in the beta band.

The primary hypothesis of this study is to show that cerebellar activity reflective of temporal sensory expectations is informative to behavior. An exploratory subhypothesis is that the thalamus will be connected to the cerebellum at the functional level.

To operationalize these hypotheses, we needed a paradigm contrasting temporal expectations that would both elicit differences in behavioral performance and in cerebellar activity. We adapted the passive paradigm of Andersen and Dalal (2021), with which we showed that cerebellar activity in the beta band (14–30 Hz) elicited by unexpectedly omitted stimulations correlates with the precision of temporal expectations. In making the paradigm active, we had participants decide whether or not at-threshold electrical stimulation was applied to their finger or whether stimulation was omitted, following a train of either precisely presented suprathreshold stimulation or a train of temporally jittered suprathreshold stimulation.

We thus expect for the primary hypothesis that (1) the finding that cerebellar activity in the beta band (14–30 Hz) for unexpectedly omitted stimulations correlating with the precision of temporal expectations will replicate; (2) participants will detect at-threshold stimulation at a higher rate when these stimulations are preceded by trains of precisely presented suprathreshold stimulation as compared with when they are preceded by trains of temporally jittered suprathreshold stimulation; and (3) participants’ detection performance will correlate with cerebellar activity evoked by the at-threshold stimulation.

In terms of the subhypotheses, we expect that the cerebellum will show differential patterns of functional connectivity to the thalamus for the at-threshold stimulation and omissions at the end of trains of stimulation, depending on whether these are precisely presented or temporally jittered.

## Materials and Methods

### Participants

Thirty right-handed, healthy participants volunteered to take part in the experiment (22 male and eight female; mean age, 29.6 years; standard deviation, 6.3 years; range, 18–43 years). The experiment was advertised in a local database that participants previously registered to. The experiment was approved by the local institutional review board in accordance with the Declaration of Helsinki. The participants provided informed consent and were compensated with 400 DKK for their participation. Two participants were excluded, one due to aborting the experiment out of discomfort and one due to excessive noise that led to rejection of >66% of the trials.

### Stimuli and procedure

Tactile stimulation was generated by two ring electrodes driven by an electric current generator (Stimulus Current Generator, DeMeTec). The ring electrodes were fastened to the tip of the right index finger. One was placed 10 mm below the bottom of the fingernail and the other one 10 mm below that. Stimulation was applied in sequences of six stimuli, with an interstimulus interval of 1,497 ms, meaning that the stimulation would not lock to the 50 Hz power from the electrical environment. All pulses were 100 µs in duration. The current applied was individualized based on a staircasing procedure. The sixth stimulus was followed either by an omission of stimulation or a weak stimulation, the target stimulation. After the seventh stimulation, omitted or weak, participants were to make a response, indicating with a button press on a response box held in their left hand, as to whether or not they were stimulated, allowing a categorization of their responses into hits, misses, false alarms, or correct rejections (MacMillan, 2002). The next sequence would begin 1,497 ms after their button press.

**Figure 1.**
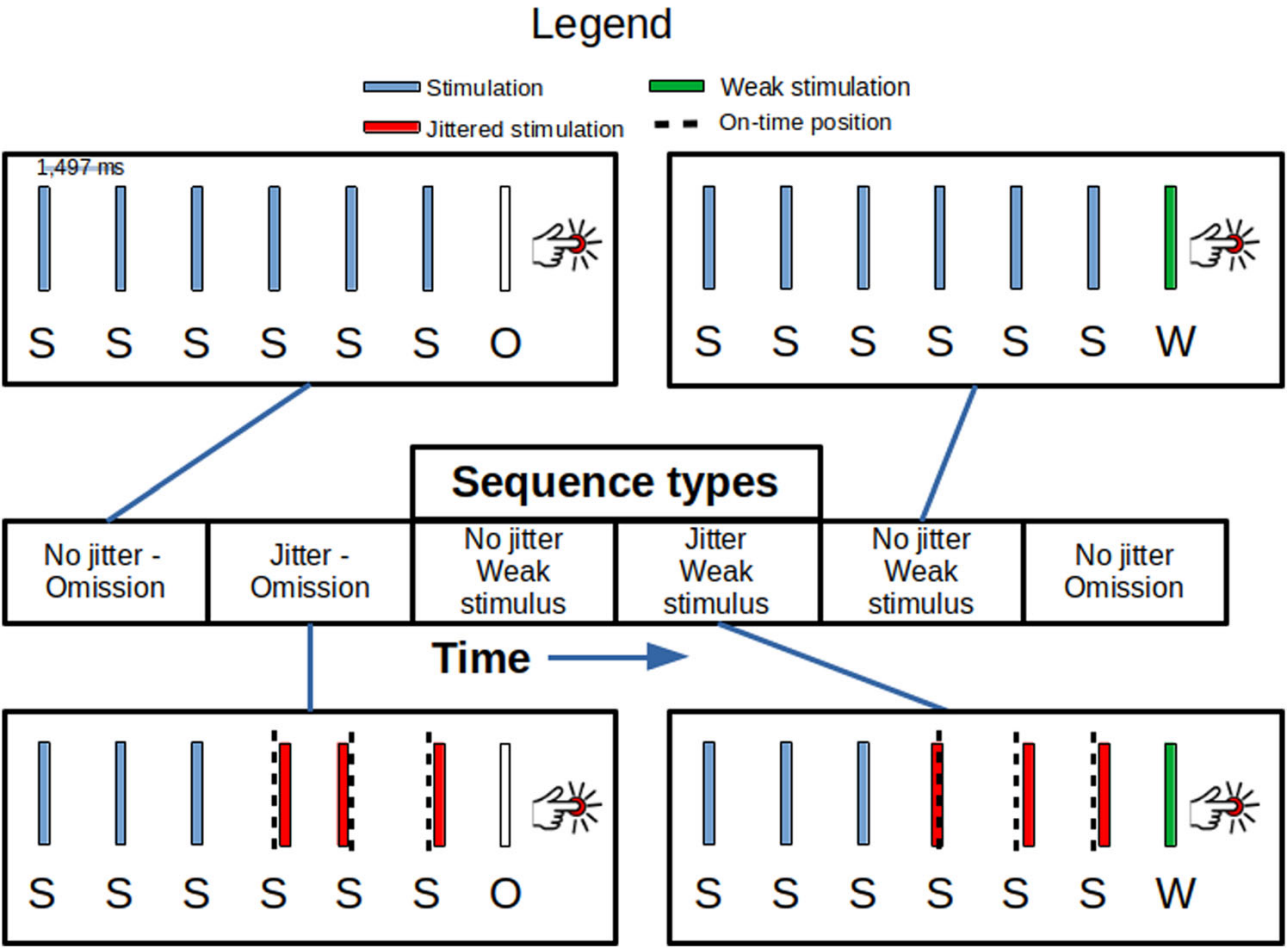
The paradigm: four kinds of sequences were administered in a pseudorandom and counterbalanced order. The first three stimulations (S) were always on time, spaced 1,497 ms apart. In the jittered sequence, up to 225 ms of jitter in either direction (forward or backward in time) was applied to the following three stimulations. The red S-bars indicate jittered stimulations and the dashed black vertical lines indicate when the stimulation should have occurred, had the sequence been regular. At the end of the sequence, either an omission (O) or a weak (W) at-threshold stimulation would occur. Participants then had to indicate with a button press whether or not they had been stimulated at the end of the sequence. For the sigmoids of the individuals related to finding the current for Weak Stimulation, please see Extended Data Figure 1-1.

The six stimuli leading up to the target stimulation were either presented in a no-jitter sequence or a jittered sequence. In the no-jitter sequence, all six stimuli were exactly 1,497 ms apart (0% jitter). In the jittered sequence, the fourth, fifth, and sixth stimuli had a jitter of up to 15% applied to them, that is, they happened from −225 to 225 ms (in steps of 1 ms) relative to when the stimulus would have occurred, had the sequence been nonjittered. For each jittered stimulation, the jitter, an integer number of milliseconds, was chosen randomly from a uniform distribution. The number of sequences ending in either weak or omitted stimulations was counterbalanced such that an equal number of sequences was applied for each of the four possible sequences [(1) nonjittered ending with an omission, (2) nonjittered ending with a weak stimulation, (3) jittered ending with an omission, (4) jittered ending with a weak stimulation; Fig. 1].

10.1523/ENEURO.0070-24.2024.f1-1 Figure 1-1 **Estimated group and subjects sigmoids:** grey lines show the estimated subject sigmoids, and the black full line shows the estimated average sigmoid; the black dot shows the proportion correct associated with the mean target current applied for weak stimulations across participants (3.8  mA). The red dots show the target current applied for the weak stimulations for each participant and the proportion of correct responses associated with that current on the staircase. The horizontal line shows the chance level during the staircasing procedure. Download Figure 1-1, TIF file.

Three hundred stimulation sequences were administered, 75 of each type presented in a pseudorandom order. This structure resulted in 300 First Stimulations, 300 Second Stimulations, and 300 Third Stimulations, due to the first three stimulations being identical between the sequences. Furthermore, this resulted in 2 × 150 Fourth Stimulations, Fifth Stimulations, and Sixth Stimulations, 150 for the nonjittered sequence, and 150 for the jittered sequence. This structure finally gave to rise 2 × 75 Omitted Stimulations (Omission 0 and Omission 15) and 2 × 75 Weak Stimulations (Weak 0 and Weak 15), 75 of each for the nonjittered sequence and 75 each for the jittered sequence. The experiment consisted of 12 blocks—between each block, verbal contact was initiated to ensure that the participant was comfortable. PsychoPy3 (version 2020.2.3; Peirce et al., 2019) was used to deliver the stimuli from a Linux workstation running Ubuntu MATE (kernel version: 4.15.0-154-generic). Electro-oculography, electrocardiography, and electromyography were recorded to detect eyeblinks, eye movements, the heartbeat, and muscle activity over the splenius muscles. For exploratory purposes, respiration was measured using a respiratory belt (MPXV4006; PI-Products) for the last 22 participants.

### Preparation of participants

In preparation for the measurement, each participant had two pairs of electrodes placed horizontally and vertically around the eyes. The heartbeat was measured by having a pair of electrodes on each collarbone. Two pairs of electrodes were placed on either side of the splenius muscles. Four head-position indicator coils, two behind the ears and two on the forehead, were placed on the participants. The ground electrode was placed on the right wrist. We subsequently digitized the head shape of each participant using a Polhemus FASTRAK (Colchester, Vermont, USA). Three fiducial points, the nasion, and the left and right preauricular points were marked alongside the positions of the head indicator coils. Furthermore, we acquired ∼150 extra points digitizing the head shape of each participant. Participants were subsequently placed in the supine position of the MEG system, and we took great care in making sure that they would lie comfortably, thus preventing neck tension. The ring electrodes were then put on the participant's finger and the respiratory belt around their abdomen. Before beginning the actual experiment, two sessions were run, a staircasing session, and a detection session. The staircasing session was a two-alternative forced choice session, where first the number 1 was shown on the screen and then the number 2 was shown, 1,497 ms between them. Either the number 1 or the number 2 was accompanied by an electrical current. A response prompt appeared on screen reminding them to answer. The participant then had to indicate with a button press when they were stimulated, either at the first or second position. Forty stimuli were applied. Each time they answered correctly, the current became less intense and vice versa more intense each time they answered incorrectly. A psychometric curve was estimated from the 40 trials and the current estimated to be equivalent to answering correct on 85% of the trials were chosen as the value for the weak stimulation in the actual experiment. The current applied in target trials (Weak) was on average 3.8 mA (SD = 1.0 mA). The current applied for the remaining stimulations was two times that of the target trials.

Subsequently, a detection session was run with 50 trials in which they were stimulated with the individualized, staircased current on approximately half the trials while no stimulation occurred on the other trials. Using the response box, they had to indicate whether or not they perceived a stimulus. If a participant ended up having more misses than hits in this session, the staircase and the detection sessions were run again before going on to the actual experiment. A response prompt appeared reminding them to answer.

In the main experiment, participants were instructed to focus on a shown fixation cross and not move their bodies or heads. No response prompt occurred during the main experiment (to prevent eliciting visual responses). Participants were therefore instructed to count the first six stimuli internally before providing the appropriate response, as to whether a weak or omitted stimulation was presented.

### Acquisition of data

MEG data were recorded on an Elekta Neuromag TRIUX system inside a magnetically shielded room (Vacuumschmelze Ak3b) at a sampling frequency of 1,000 Hz. Data were acquired with low-pass and high-pass filters applied, at 330 and 0.1 Hz respectively.

### Processing of MEG data

For each participant, we inspected the raw data traces and their power spectra densities for bad sensors. Sensors were removed if they showed a consistently higher or lower power than the average. We analyzed both evoked and oscillatory responses.

**Figure 2.**
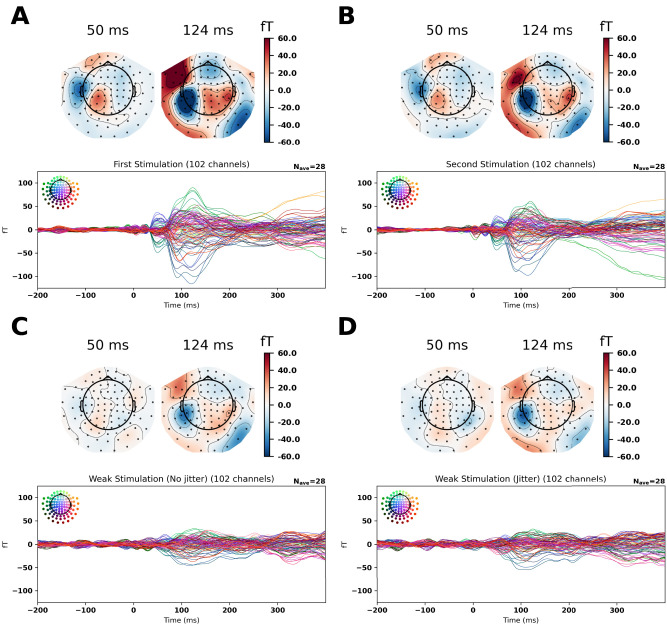
Evoked responses for the stimulation data: the first and second stimulations are shown (***A***,***B***) revealing topographies compatible with primary (50 ms) and secondary (124 ms) somatosensory cortical activation. The weak stimulations are also shown (***C***,***D***), nonjittered and jittered. Only the secondary somatosensory cortical activation is apparent and weaker than for the first and second stimulations. For all topographies related to secondary somatosensory cortical activation (124 ms), posterior contributions are also seen. Bad channels have been interpolated.

All MEG analyses were carried out in MNE-Python (version 1.3.1; Gramfort et al., 2013 https://doi.org/10.5281/zenodo.7671973). To preserve the rank of the data, MaxFilter was not applied. Signal space projections reflecting the general environmental noise of the recording environment were projected out for the sensor space analyses (Fig. 2) but were not applied for source space analyses.

For the analysis of evoked responses, we low-pass filtered the data: 40 Hz [one-pass, noncausal, finite impulse response; zero phase; upper transition bandwidth: 10.00 Hz (−6 dB cutoff frequency: 45.00 Hz; filter length 331 samples; a 0.0194 passband ripple and 53 dB stopband attenuation)]. We subsequently segmented the filtered data into epochs of 1,200 ms, 200 ms prestimulus and 1,000 ms poststimulus. The epochs were demeaned using the mean value of the prestimulus period. Bad channels were removed from each participant's recording based on inspection of data. Four participants had one magnetometer removed, and six participants had two magnetometers removed. For the sensor space analysis, segments of data including magnetometer responses >4 pT (peak-to-peak) or gradiometer responses >400 pT/cm (peak-to-peak) were rejected. Subsequently epochs were averaged to create evoked responses for each of the trial types. On average for each trial the following amounts of trials were kept after rejection: First Stimulation, 291 (SD = 21.2); Second Stimulation, 293 (SD = 18.2); Weak, non-jittered, 74 (SD = 3.35); Weak, jittered, 74 (SD = 2.20); Omission, nonjittered, 74 (SD = 3.93); and Omission, jittered, 74 (SD = 3.53). For the source space analysis, where signal space projections were not projected out, we did not apply this automatic rejection procedure, as the strong influence of environmental noise would mean that they would all be rejected. EOG was not used to reject epochs since beamforming suppresses ocular artifacts well (Sekihara et al., 2004). These procedures area in line with recent advice for how to analyze electrophysiological data (Delorme, 2023).

For the analysis of beta band responses, the data were bandpass filtered [14–30 Hz; lower transition bandwidth: 3.50 Hz (−6 dB cutoff frequency: 12.25 Hz); upper transition bandwidth: 7.50 Hz (−6 dB cutoff frequency: 33.75 Hz); filter length 943 samples; passband ripple: 0.0194; stopband attenuation: 53 dB)]. This filter was one-pass, noncausal, finite impulse response and zero phase. We subsequently Hilbert transformed the filtered data and segmented it into epochs of 1,500 ms, 750 ms prestimulus and 750 ms poststimulus.

#### Source reconstruction

A linearly constrained minimum variance beamformer was used to reconstruct sources for both the evoked and the oscillatory responses. For the evoked responses, the data covariance matrix was estimated based on the poststimulus time period (from 0 to 1,000 ms) and for the oscillatory responses the data covariance matrix was based on the whole period (from −750 to 750 ms). Only magnetometers were used as these are the most sensitive to deeper sources. No regularization was applied to the covariance matrices as they were well-conditioned. For estimating the spatial filter, we used the unit-noise-gain constraint, as this results in the best specificity for estimating the source time courses (Sekihara and Nagarajan, 2008), choosing the source orientation that maximizes the signal-to-noise ratio. For both evoked and oscillatory responses, we took the absolute value of the source time courses.

To create a source space where the source time courses would be estimated from, we acquired sagittal T1-weighted 3D images for each participant using a Siemens Magnetom Prisma 3 T MRI. The pulse sequence parameters were as follows: 1 mm isotropic resolution; field of view, 256 × 240 mm; 192 slices; slice thickness, 1 mm; bandwidth per pixel, 290 Hz/pixel; flip angle, 9°; inversion time (TI), 1,100 ms; echo time (TE), 2.61 ms; and repetition time (TR), 2,300 ms. We furthermore acquired T2-weighted 3D images. The pulse sequence parameters were as follows: 1 mm isotropic resolution; field of view, 256 × 240 mm; 96 slices; slice thickness, 1.1 mm; bandwidth per pixel, 780 Hz/pixel; flip angle, 111°; echo time, 85 ms; and repetition time, 14,630 ms. Based on the T1 images, a one-layered boundary element method volume conductor was modeled based on delineating the inner skull using the watershed algorithm, as implemented in FreeSurfer. Based on the segmentation of the brain, a volumetric source space was created with sources 7.5 mm apart. After the estimation of the source time courses in the individual source space, the source time courses were morphed onto *fsaverage*, a template from FreeSurfer (Dale et al., 1999; Fischl et al., 1999). All regions of interest were defined using the Automated Anatomical Labeling (AAL) atlas (Tzourio-Mazoyer et al., 2002) except for secondary somatosensory cortex, which was defined using the Harvard Oxford cortical atlas (Desikan et al., 2006).

### Statistical analysis

#### Behavioral data

For the statistical analysis of the behavioral data, mixed effects models (Gelman and Hill, 2006) were fitted. We fitted logistic regression models with Accuracy as a binary dependent variable. For the final stimulation in a sequence, accuracy was coded as 1 if the participant indicated that they were stimulated, when they were in fact stimulated or if the participant indicated that they were not stimulated when they were in fact not stimulated. Accuracy was coded as 0 if the participant indicated that they were stimulated, when they were in fact not stimulated or if the participant indicated that they were not stimulated when they were in fact stimulated.

We had two independent variables of interest, Stimulation Type and Stimulation Variance. Stimulation Type was a factorial variable with two levels, Omission or Weak. Stimulation Variance was a continuous variable expressing the variance around the expected time of stimulation for the jittered stimulations, that is, the fourth, fifth, and sixth stimulations. The Stimulation Variance was calculated as follows:

(1) $$\begin{array}{c} \sigma_{\mathrm{STIMULATION}}^{2}=\overset{n\;=\;6}{\underset{i\;=\;4}{\sum }}\;{\left(t_{\mathrm{ACTUAL}}^{\left(i\right)}-t_{\mathrm{EXPECTED}}^{\left(i\right)}\right)}^{2}\\ \mathrm{with}:\;t_{\mathrm{EXPECTED}}^{\left(i\right)}=1,497\;\mathrm{ms}\times i \end{array},$$

*t*_ACTUAL_ was when the stimulation actually happened. This resulted in a Stimulation Variance equal to zero for the nonjittered sequences and a variable Stimulation Variance for the jittered sequences.

For the subject-level effects (random effects), we modeled a unique intercept for each subject and two unique slopes of the Stimulation Variance as dependent on the Stimulation Type, Omission or Weak. For the group-level effects (fixed effects), we followed the strategy of beginning with a null model that only estimated a mean accuracy (an intercept). Subsequently, we added the independent variables of interest in a step-wise fashion to the model—for each step calculating the likelihood-ratio between the two models:

(2) $$\mathrm{Likelihoodratio}=2(l(m_{2})-l(m_{1})),$$

with *l* as the log-likelihood and *m* as the models (*m*_1_ is nested within *m*_2_). This ratio is approximated by a *χ*^2^ distribution. Whether the null hypothesis that the likelihood ratio is 0 could be rejected was evaluated using a *χ*^2^ test with the likelihood ratio as the test statistic and degrees of freedom equal to the difference in free parameters between the models. We rejected the null hypothesis at *α* = 0.05.

The mixed models were estimated using the *lme4* package (Bates et al., 2014) from *R* (R Core Team, 2020).

#### MEG data

For the statistical analysis of the MEG data, we focused on the regions and time intervals reported by Andersen and Dalal (2021), that is, the cerebellum, and the primary and secondary somatosensory areas. We used a linearly constrained minimum variance beamformer (Sekihara and Nagarajan, 2008) to extract time courses from each of these areas.

For evoked responses, the trial type comparisons of interest were between First Stimulation and Second Stimulation, between Weak, no jitter and Weak, jittered, and between Omission, no jitter and Omission, jittered in the time interval between 0 and 150 ms. Two time courses were extracted, namely, the ones that showed the maximal response for each condition within the region of interest. We ran cluster permutation statistics on the mean of the extracted time courses with 10,000 permutations (Maris and Oostenveld, 2007), using *α* = 0.05.

For the beta band (14–30 Hz) oscillatory responses of the omissions, the trial type comparisons of interest were the same. The time interval run on was 0–200 ms based on the findings of Andersen and Dalal (2021, 2022). In a separate analysis in the beta band, we investigated the contributions of thalamus as a potential contributor to stimulus processing, as this was also found by Andersen and Dalal (2021, 2022).

#### Correlations between behavioral and MEG data

To estimate correlations between brain activity and behavior, we, based on the winning logistic regression model, extracted the variance-related slope for each participant for each task condition, that is, for Weak and Omission. These were then to be correlated with relevant brain activity. The more negative the slope, the more a given participant's performance would deteriorate as a function of the variance introduced in the jittered conditions. Spearman correlations were run as the data was non-normal.

#### Envelope correlations

To investigate oscillation-mediated connectivity between regions of interest, we used envelope correlation (Hipp et al., 2012) that as its name indicates estimates the correlation of envelopes of oscillatory activity. We defined the cerebellum as a seed and investigated its connections to the rest of the brain and the thalamus in particular. We conducted an analysis of variance using two factors, Stimulation Type (two levels: weak, omission) and Regularity (two levels: nonjittered, jittered). We investigated the envelope correlations from −100 to 100 ms around Weak and Omission in the beta band. This was done as an exploratory analysis.

## Results

### Behavior

**Figure 3.**
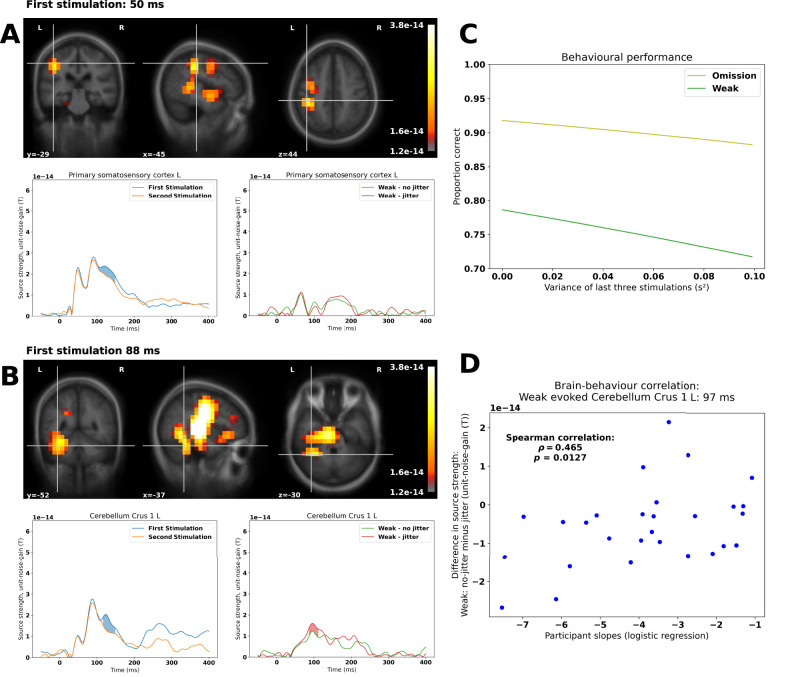
Source localization, behavioral performance, and brain–behavior correlation: ***A***, Primary somatosensory activation; for the first and second stimulations, we find a primary somatosensory activation at 50 ms. We find a difference cluster around the last peak (blue fill). The first response for the weak stimulations is at 60 ms. ***B***, Cerebellar activation; for the first and second stimulations, we find three cerebellar peaks—at 49, 87, and 124 ms, respectively. We find a difference cluster around the last peak (blue fill). For the weak stimulations, we only find one peak, 97 ms with a difference cluster (red fill). The vertices are chosen by averaging the maximally responding vertices for First Stimulation and Second Stimulation and for Weak Stimulation (nonjittered) and Weak Stimulation (jittered), respectively, within the relevant region of interest. ***C***, Proportion correct as dependent on the variance of the last three stimulations. ***D***, Correlation between the estimated participant slopes based on multilevel logistic regression and the difference in estimated peaks for the cerebellum for the Weak Stimulations (no-jitter minus jitter); we find a significant positive correlation between the estimated participant slopes and the difference between the evoked responses to the Weak Stimulations. For the difference waves for the evoked responses with standard errors of the mean, please see Extended Data Figure 3-1. For the analyses done with cerebellar lobule VI instead with cerebellar crus I, please see Extended Data Figure 3-2.

Adding Stimulation Type as a group-level variable to the null model, which only modeled mean accuracy besides the subject-level effects, resulted in a significant increase in the likelihood of the model, *χ*^2^(1) = 13.8, *p* = 0.0020. Further adding Stimulation Variance to this model also resulted in a significant increase in the likelihood of the model, *χ*^2^(1) = 5.92, *p* = 0.014. Adding the interaction between Stimulation Type and Stimulation Variance did, however, not result in a significant increase in the likelihood of the model, *χ*^2^_(1)_ = 0.0083, *p* = 0.93. Our winning model thus includes group-level effects of Stimulation Variance and Stimulation Type, but no interaction between them. Given this model, the estimated group-level accuracy when Stimulation Variance was 0 (no jitter) for Omission was 91.7% and for Weak it was 78.7% (Fig. 3*C*). At the mean Stimulation Variance of the jittered trials (0.032 s^2^), the estimated group-level accuracy had dropped to 90.7% for Omission and to 76.5% for Weak. At the maximum Stimulation Variance (0.09 s^2^), the estimated group-level accuracy had dropped to 88.4% for Omission and to 71.6% for Weak (Fig. 3*C*). We can thus conclude that our paradigm had the intended effect on behavior, resulting in detection of stimuli becoming worse, the more jitter there was in the stimulations leading up to the target stimulation.

10.1523/ENEURO.0070-24.2024.f3-1 Figure 3-1 **Evoked differences in the cerebellum and the primary somatosensory cortex:** The blue lines show the differences in source strength for the sources presented in Figure 3. The shaded areas indicate the standard error of the mean. Download Figure 3-1, TIF file.

**Figure 4.**
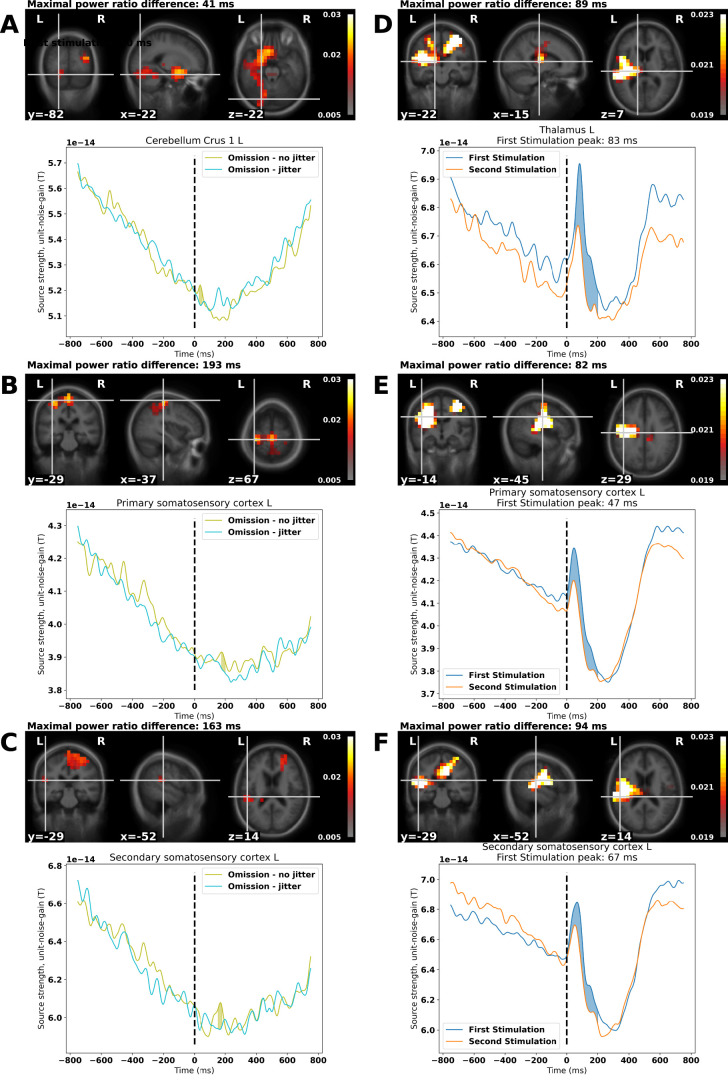
Beta band (14–30 Hz) results: ***A***, cerebellar crus I shows increased omission-related activity just after the time of the expected stimulation for nonjittered omissions compared with jittered omissions. ***B***, ***C***, Furthermore, primary and secondary somatosensory cortices are also involved with secondary somatosensory cortex showing involvement after that of cerebellar crus I and primary somatosensory cortex. ***D–F***, For the brain maps, the maximal ratio differences in beta band power between First Stimulation and Second Stimulation are shown for the thalamus and the primary and secondary somatosensory cortices. Note however that the individual peaks differ in their timing, peaking at 83, 47, and 67 ms, respectively. The time courses are extracted from the voxel shown in the brain maps and the solid filling show the temporal extent of the cluster. In the brain maps, only voxels part of a cluster are shown, all other voxels are masked. Voxels show the power change either between nonjittered Omissions and jittered Omissions or between First Stimulation and Second Stimulation (*x*_1_ − *x*_2_) / (*x*_1_ + *x*_2_). For the ratios between conditions with standard errors of the mean, please see Extended Data Figure 4-1.

10.1523/ENEURO.0070-24.2024.f3-2 Figure 3-2 **Cerebellar Lobule VI analyses:** A: Similar to Figure 3B. B: Similar to Figure 3C. C: Similar to Figure 3B. D: Similar to Figure 3-1. E: Similar to Figure 3B. F: Similar to Figure 3-1. G: Similar to Figure 4A. H: Similar to Figure 4A. I: Similar to Figure 4-1. Download Figure 3-2, TIF file.

### MEG

#### Evoked responses

For the evoked responses, we found the expected responses compatible with primary and secondary somatosensory cortical activations (Fig. 2). The topographies related to the secondary somatosensory activation also included posterior contributions, potentially compatible with cerebellar contributions. The linearly constrained minimum variance beamformer revealed the expected primary and secondary somatosensory cortical activations for First Stimulation. Furthermore, an activation covering left cerebellar lobule VI and left cerebellar crus I was also revealed (Fig. 3). We used the coordinates associated with the maximum activation for each of these regions of interest to estimate the responses associated with Weak, nonjittered and Weak, jittered. Running the cluster permutation statistics revealed six significant effects of condition. The first four ones were for the comparison between First Stimulation and Second Stimulation in the primary somatosensory cortex, *p* = 0.0004; secondary somatosensory cortex, *p* = 0.0001; left cerebellar lobule VI, *p* = 0.0088 (Extended Data Figure 3-2); and left cerebellar crus I, *p* = 0.0084. The clusters associated with this extended from 106 to 149 ms, from 90 to 142 ms, from 116 to 149 ms, and from 116 to 149 ms, respectively. The two last ones were for the comparisons between Weak, nonjittered and Weak, jittered in the left cerebellar lobule VI, *p* = 0.0124 (Extended Data Figure 3-2) and the left cerebellar crus I, *p* = 0.0295. The clusters associated with this extended from 85 to 113 ms and from 87 to 111 ms, respectively. The differences between source time courses and their associated standard errors of the mean can be seen in Extended Data Figure 3-1.

##### Brain–behavior correlations—evoked responses

We correlated the participant level slopes for the Weak Stimulation condition, estimated from the behavioral model, with the differences (between Weak, no jitter and Weak, jittered) in peak evoked cerebellar crus I activity (97 ms; Fig. 3*B*), *ρ*_26_ = 0.465, *p* = 0.0127 (Fig. 3*D*). [Cerebellar lobule VI showed a similar effect, *ρ*_26_ = 0.374, *p* = 0.0497 (Extended Data Figure 3-2).]

The positive sign of *ρ* indicates that stronger cerebellar activation to a nonjittered weak stimulus than to a jittered weak stimulus was correlated with better performance.

#### Oscillatory responses

##### Involvement of the beta band (14–30 Hz) in prediction

Running the cluster permutation statistics, we reject the null hypothesis that the nonjittered omissions and jittered omissions are exchangeable, *p* = 0.0127. We replicate the finding from Andersen and Dalal (2021, 2022), as we find a cluster (Fig. 4*A*) that contains left cerebellar lobule VI extended from 30 to 65 ms and from 98 to 115 ms (Extended Data Figure 4-1) and left cerebellar crus I, extended from 29 to 52 ms. This cluster also includes the left putamen (Fig. 4*C*). We also find a cluster that includes left primary somatosensory cortex, (Fig. 4*B*; 176–200 ms). Finally, we find a cluster that includes left secondary somatosensory cortex (Fig. 4*C*; 154–179 ms) as well as the frontal pole.

10.1523/ENEURO.0070-24.2024.f4-1 Figure 4-1 **Beta band (14-30  Hz) ratios for omissions and stimulations respectively:** The blue lines indicate the ratio (x_1_-x_2_)/(x_1 _+ x_2_) for omissions (left column), no jitter and jitter, and stimulations (right column), first and second. The shaded areas indicate the standard error of the mean. Download Figure 4-1, TIF file.

For the comparison between First Stimulation and Second Stimulation, we reject the null hypothesis that they are exchangeable, *p* = 0.000977. Peaks are found in the primary and secondary somatosensory cortices and the thalamus (Fig. 4*D–F*). These are separated in time, supporting their veracity. The thalamic peak is explored further below, “Is it really the thalamus?”. The ratios between responses and their associated standard errors of the mean are shown in Extended Data Figures 3-2 and 4-1.

### Envelope correlations

**Figure 5.**
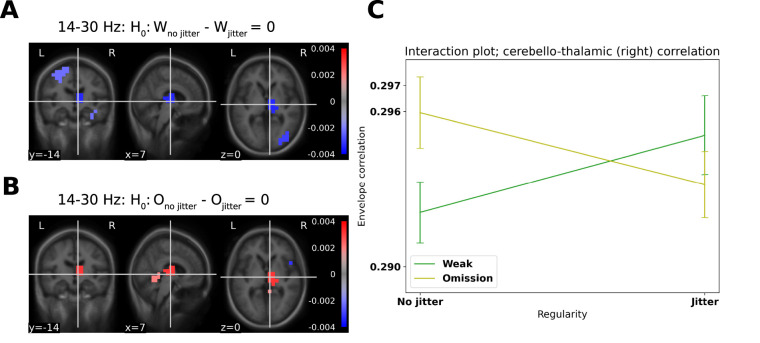
Cerebello-thalamic envelope correlations: envelope correlation using left cerebellar crus I as a seed; only envelope correlations in the beta band (14–30 Hz) that are significant at *α* = 0.05 are shown. ***A***, Cerebello-thalamic envelope correlations are stronger for the Weak, nonjittered condition relative to the Weak, jittered condition, whereas ***B***, Cerebello-thalamic envelope correlations are stronger for the Omission, nonjittered condition relative to the Omission, jittered condition. ***C***, Interaction plot for the right thalamus, highlighting the increase in correlation for weak stimulations when introducing jitter and the decrease in correlation for omitted stimulations when introducing jitter. Error bars are standard errors of the mean.

The envelope correlations in the beta band revealed differences in correlations for jittered and nonjittered weak stimulations and omitted stimulations between left cerebellar crus I and the right thalamus. We here focus on the cerebellar crus I, as it showed the strongest correlation with behavior. An analysis of variance revealed an interaction between Stimulation Type (2 levels) and Regularity (2 levels), *F*_(1,100)_ = 4.42, *p* = 0.0376 for the right thalamus (Fig. 5).

Beta band envelopes in cerebellar crus I correlated differently with the right thalamus dependent on whether the last stimulus was omitted or weak, while this in turn depended on whether or not the sequence was jittered. Specifically, we found that cerebello-thalamic envelope correlations increased for Weak Stimulations when going from nonjittered to jittered, whereas these decreased *for* Omitted Stimulations when going from nonjittered to jittered (Fig. 5*C*).

### Is it really the thalamus?

**Figure 6.**
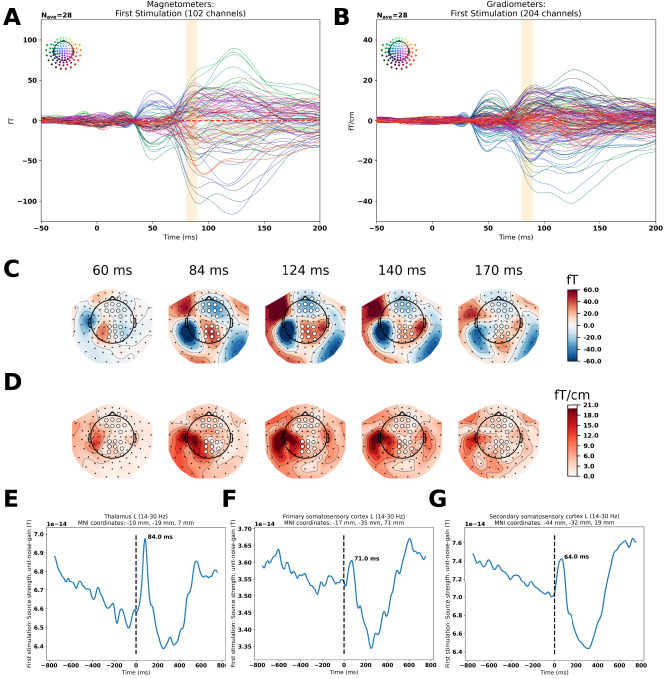
Compatibility of the grand averaged evoked response with the beta band (14–30 Hz) thalamic response found. ***A***, Magnetometers: a component is highlighted at 84 ms for the first stimulations for the evoked responses (not discussed in Fig. 2). ***B***, Planar gradiometers: the same component is highlighted at 84 ms. ***C***, The topographical plots for the magnetometers are consistent with two underlying sources at 84 ms, one lateral and one central, highlighted manually with white dots; at 124 ms they are still both present, whereas at 140 ms the lateral one starts dominating and at 170 ms, it is the only one that remains. ***D***, On the topographical plots for the gradiometers, the central component is absent, consistent with a deep central source in ***C*** (same sensor triplets highlighted in white). ***E***, The peak of the beta band response localized to the left thalamus match the peaks in the evoked response (***A***,***B***). ***F***, The beta band response of the primary somatosensory cortex peaking earlier, 71 ms, than the thalamic response. ***G***, The beta band response of the secondary somatosensory cortex peaking earlier, 64 ms, than the thalamic response. The Montreal Neurological Institute (MNI) coordinates for these estimated sources were chosen based on finding central coordinates for the relevant regions from the MNI brain from FSL (Smith et al., 2004).

To assess whether the thalamic findings could be due to the smearing of activity from other areas, we investigated the evoked responses and the source reconstructions more closely (Fig. 6). We find evidence of a peak in the sensor space related to a deep source, since it shows clearly on the magnetometers and less so on the gradiometers (Fig. 6*A*,*D*). Its timing (Fig. 6*E*) is also different from the other expected sources of the somatosensory cortex (Fig. 6*F*,*G*).

## Discussion

### Brief summary of the results

In summary, we found that the detection of weak stimulation was affected by the jitter introduced into stimulation sequences (Fig. 3*C*). In parallel, we found cerebellar responses evoked by all of the stimulation sequences (Fig. 3*B*). Specifically, for the weak stimulations, the difference in estimated source current density between jittered and nonjittered conditions correlated with the scale of the slope indicating how much participant performance was affected by variation in the stimulation sequences (Fig. 3*D*). In the beta band, we furthermore found differences in the primary and secondary somatosensory cortices and the thalamus between the first, surprising, stimulation and the second, expected, stimulation (Fig. 4*D–F*). Finally, using the cerebellum as a seed we found that the envelope of cerebellar crus I correlated with the envelope of right thalamic activity (Fig. 5*A*,*B*).

The evoked cerebellar responses here are contralateral to the stimulation, consistent with how the underlying sensory fibers project (Manni and Petrosini, 2004). Functional magnetic resonance imaging studies also show contralateral responses of the cerebellum to touch of the hand and the foot (Gao et al., 1996; Bushara et al., 2001; Takanashi et al., 2003), as does the expectation-related differences in omissions found in the present study (Fig. 4*A*; see also Andersen and Dalal, 2021). This suggests that the relevant pathways for expectation-related activity indeed also project contralaterally despite ipsilateral dominance for several cerebellar pathways such as the movement pathways (Grodd et al., 2001).

### The functional relevance of cerebellar timing activity

The variance introduced in the behavioral task in the jittered condition makes it harder to detect the weak stimulations, which are otherwise identical. This indicates that there must be an internal mechanism that tracks the expected timing of stimulation (Fig. 3*C*). The replication of the beta band finding that cerebellum predicts the timing of expected stimulation corroborates the findings of Andersen and Dalal (2021, 2022) and also highlights the fact that it can be done with half the trials (75 instead of 150; Fig. 4*A*). This establishes beta band cerebellar activity as following expected patterns of stimulation and establishes cerebellum as a candidate for enabling the better performance seen when stimulation sequences are regular.

That the proposed cerebellar clocking activity has functional relevance is evidenced by the correlation (Fig. 3*D*) found between differences in cerebellar evoked responses (Fig. 3*B*) for weak stimulations depending on whether they are preceded by a jittered sequence or not. Interestingly, the strength of the cerebellar evoked response does not enable stronger performance on the behavioral task, but the opposite: the stronger the cerebellar response to the jittered weak stimulation was relative to the nonjittered one, the worse subject performance was, as estimated by the slope of their individual fits. This is evidence that the cerebellum is not merely encoding the perceived magnitude of the electrical stimulation—on the other hand, the increased cerebellar response for the jittered sequences may indicate inhibition of weak stimulation following a jittered sequence. The less inhibited a weak stimulation was, that is, the smaller the difference between weak cerebellar evoked responses (Fig. 3*D*; *y*-axis), the greater the probability was of detecting it (Fig. 3*C*). In fact, the Purkinje cells, which are likely the neurophysiological basis of MEG signals from the cerebellum (Okada et al., 1987), have an inhibitory role in sensory processing and perception (Ito et al., 1970; D’Angelo et al., 2009; D’Angelo, 2018). The role of this particular mechanism could be to filter away nonsalient crossings of the sensory threshold, signaling whether a stimulation took place or not. According to this interpretation, weak stimulation following a jittered sequence would have a higher chance of being filtered away, thereby leading to more misses as variance increases (Fig. 3*C*). This interpretation of cerebellar activity as inhibitory is also consistent with the impact of jitter on performance for omissions. That beta band power is lower for omissions following a jittered sequence implies that any nonsalient crossings of the threshold are less likely to be filtered away. As a consequence, more false alarms should follow as variance increases, which is indeed what we observe (Fig. 3*C*).

### A thalamic role?

We also found a difference in beta band activity between first and second stimulations—the thalamic responses are similar to the ones reported by Andersen and Dalal (2022, their Supplementary Material 3: New Figure 3). This justified running the exploratory functional connectivity analysis of the cerebellum and the thalamus, where we found an interaction in the envelope correlations between left cerebellar crus I and the right thalamus dependent on stimulation type and regularity of the preceding sequence. Assuming that this connection reflects the degree to which the cerebellar expectations are relevant to informed behavior, the overall decrease in connectivity for jittered weak stimulation compared with nonjittered weak stimulation may explain the decrease in performance in detecting weak stimulation when jitter is introduced (Fig. 3*C*). Put in terms of signal detection theory, when the number of hits decreases and thus the number of misses increases, the connectivity increases. And vice versa, when the number of correct rejections decreases, the number of false alarms increases, the connectivity decreases. That means that a decrease in connectivity between left cerebellar crus I and the right thalamus corresponds with answering stimulus-present and an increase in cerebello-thalamic connectivity answering no-stimulus-present, independently of whether a stimulus was actually present. This can be interpreted as cerebellum connecting more strongly with thalamus when there is no perceived stimulus, in line with the idea that the cerebellum detects omissions from the established pattern, which results in increased beta band power (Fig. 4*A*). This interpretation of the involvement of right thalamus in motor commands is consistent with the contralaterality of thalamic efferents (Herrero et al., 2002), that is, subjects indicated their answers with their left hand. The timing of the response may seem late, as the first-order thalamic relay-activity happens early, <20 ms. However, it has been argued that higher-order relay activity is likely to happen at later stages in the processing of stimuli, especially if they are relevant to motor output (Guillery and Sherman, 2002; Sherman, 2007).

As appealing as these thalamic and cerebello-thalamic results are, it is important to treat MEG results of thalamus with a critical attitude. The thalamus is a nonoptimal target for MEG due to its deep location toward the center of the head and the configuration of its neurons, which do not form a field as open as those of the cerebral and cerebellar cortices (Lorente de Nó, 1947). However, simulation and experimental studies support the feasibility of detecting the thalamus using MEG (Attal et al., 2009, 2012; Attal and Schwartz, 2013). Therefore, we scrutinized the thalamic responses more carefully (Fig. 6) to make sure that the results were not merely due to a smearing of activity from nearby regions as a result of our source localization procedure. Several points are indicative of this not being the case. First, the peak thalamic response at 84 ms for the beta band (14–30 Hz; Fig. 6*E*) coincides with an evoked response at 84 ms visible in sensor space (Fig. 6*A*,*B*). Second, the topographical pattern at 84 ms for the magnetometers is compatible with a central, deep source (Fig. 6*C*; and a lateral source, the secondary somatosensory cortex). Third, the topographical pattern at 84 ms (or later) for the planar gradiometers does not include a similar, central activation, but only a lateral one (Fig. 6*D*). Fourth, neither the primary somatosensory cortex nor the secondary somatosensory cortex peaked at a similar time for the beta band (Fig. 6*F*,*G*). We do not mean to imply that this means that the 84 ms peak of thalamic beta band activity is reflective of underlying oscillations—it may be that the focusing on the beta band makes it easier to separate the potential thalamic response from the concurrently unfolding secondary somatosensory cortex evoked response (Fig. 6*A–D*), which is represented mainly at a lower frequency.

### Validity

In the current study, we cannot separate whether the cerebellar predictions we report are purely bottom-up, driven by the regularity of the stimuli, or whether they also depend on top-down mechanisms, driven by the demands of the task. We, however, report a cerebellar difference between regular and jittered sequence (Fig. 4*A*), similar to our earlier study (Andersen and Dalal, 2021), where subjects had no task, and where their attention was directed toward an unrelated movie instead. Evidence of cerebellar top-down temporal attention has also been reported though (Breska and Ivry, 2020; Craig et al., 2021).

The reliability of the reported correlations should also be mentioned (Fig. 3*D*). Marek et al. (2022) showed that many reported brain–behavior correlations are likely to be spurious and that they would require sample sizes of thousands of participants to be adequately powered. An important aspect of their analysis however was that it pertained only to brain-wide association studies—our reported correlation (*ρ* = 0.465), however, was based on the specific cerebellar finding also reported in Andersen and Dalal (2021) and replicated here (Fig. 4*A*), which was hypothesized to be functionally relevant to behavior.

### Concluding remarks

Our study shows cerebellar responses have functional relevance, as they correlate with performance in detecting weak stimuli (Fig. 3*D*). Furthermore, cerebellar beta band (14–30 Hz) responses encode predictions (Fig. 4), and connectivity results suggest that the evaluation of these cerebellar predictions inform behavior through connectivity to the thalamus (Fig. 5).

These findings highlight the need to include noncortical areas (Andersen et al., 2020; Shine et al., 2023) in our understanding of the brain and its capabilities in prediction and using the environment to inform behavior.

## Synthesis

Reviewing Editor: Alexander Soutschek, Ludwig-Maximilians-Universitat Munchen Decisions are customarily a result of the Reviewing Editor and the peer reviewers coming together and discussing their recommendations until a consensus is reached. When revisions are invited, a fact-based synthesis statement explaining their decision and outlining what is needed to prepare a revision will be listed below. The following reviewer(s) agreed to reveal their identity: Gianpaolo Demarchi. Note: If this manuscript was transferred from JNeurosci and a decision was made to accept the manuscript without peer review, a brief statement to this effect will instead be what is listed below.
