# Supplementary figures and images for: Detection of Threshold-Level Stimuli Modulated by Temporal Predictions of the Cerebellum
Source: eNeuro. 2024 Apr 23;11(4):ENEURO.0070-24.2024. doi: 10.1523/ENEURO.0070-24.2024 (PMC11064121; doi:10.1523/ENEURO.0070-24.2024)

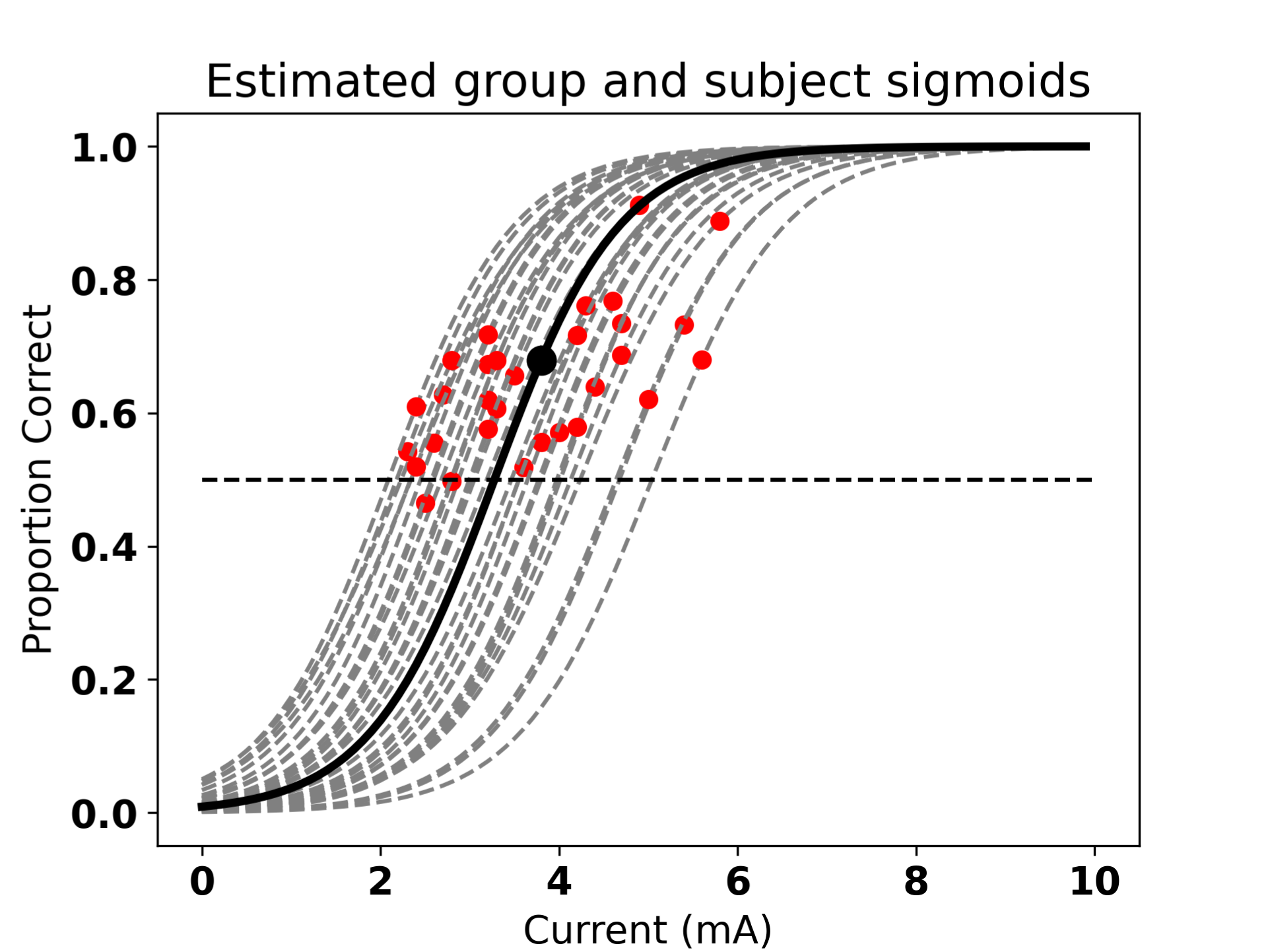

Supplement: Figure 1-1 — Estimated group and subjects sigmoids: grey lines show the estimated subject sigmoids, and the black full line shows the estimated average sigmoid; the black dot shows the proportion correct associated with the mean target current applied for weak stimulations across participants (3.8 mA). The red dots show the target current applied for the weak stimulations for each participant and the proportion of correct responses associated with that current on the staircase. The horizontal line shows the chance level during the staircasing procedure. Download Figure 1-1, TIF file. [file eneuro-11-ENEURO.0070-24.2024-s002.tif]

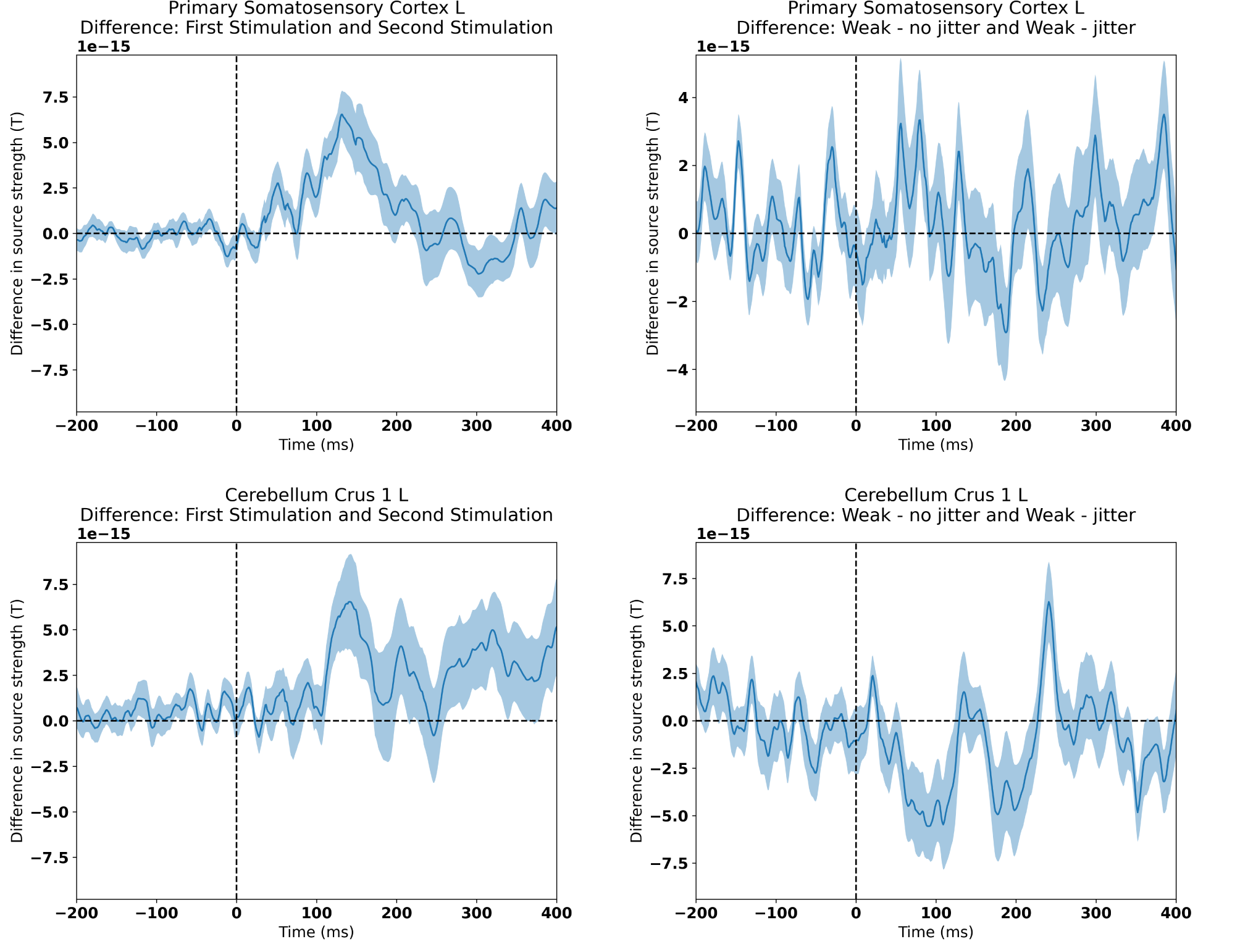

Supplement: Figure 3-1 — Evoked differences in the cerebellum and the primary somatosensory cortex: The blue lines show the differences in source strength for the sources presented in Figure 3. The shaded areas indicate the standard error of the mean. Download Figure 3-1, TIF file. [file eneuro-11-ENEURO.0070-24.2024-s003.tif]

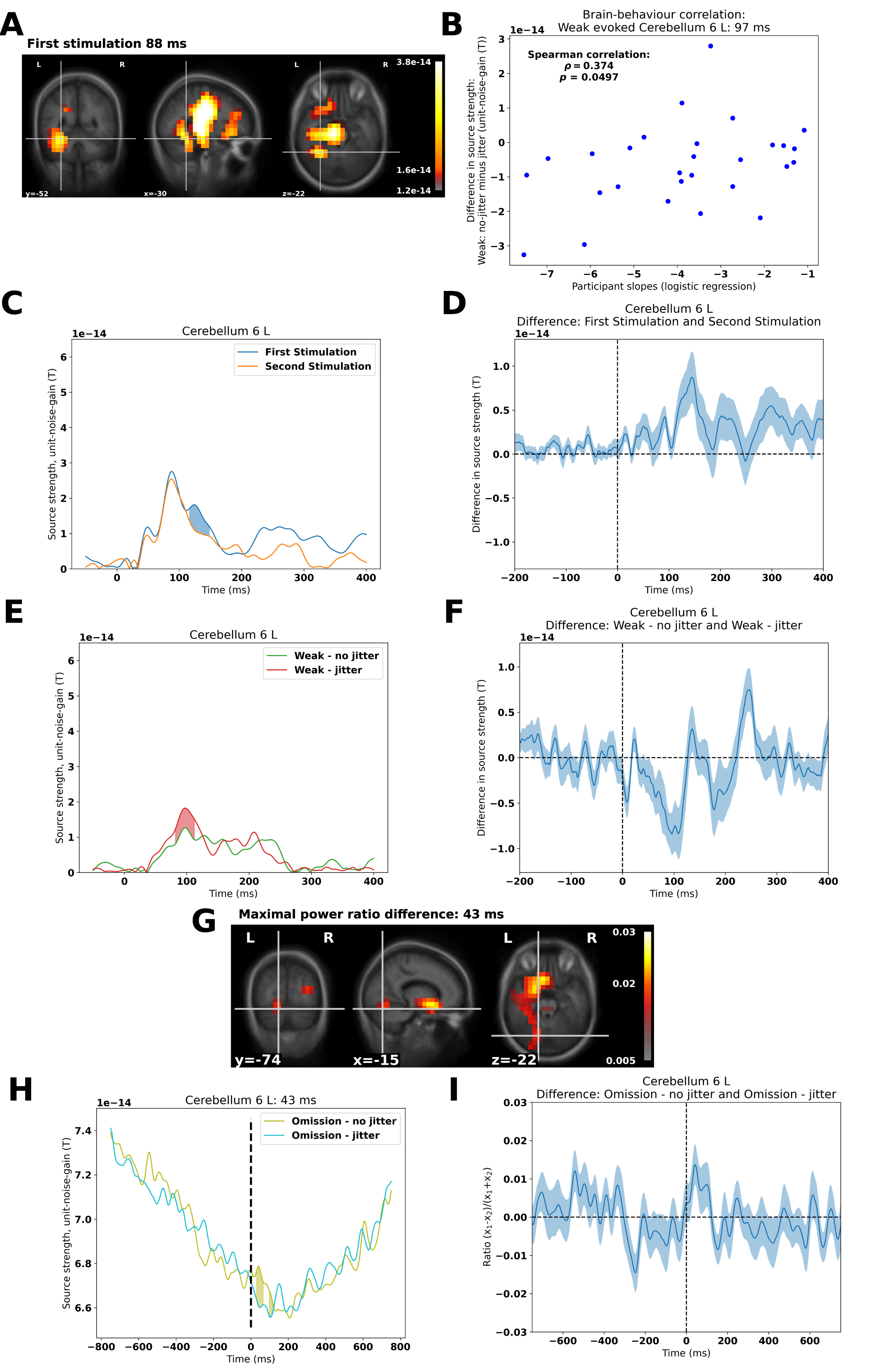

Supplement: Figure 3-2 — Cerebellar Lobule VI analyses: A: Similar to Figure 3B. B: Similar to Figure 3C. C: Similar to Figure 3B. D: Similar to Figure 3-1. E: Similar to Figure 3B. F: Similar to Figure 3-1. G: Similar to Figure 4A. H: Similar to Figure 4A. I: Similar to Figure 4-1. Download Figure 3-2, TIF file. [file eneuro-11-ENEURO.0070-24.2024-s004.tif]

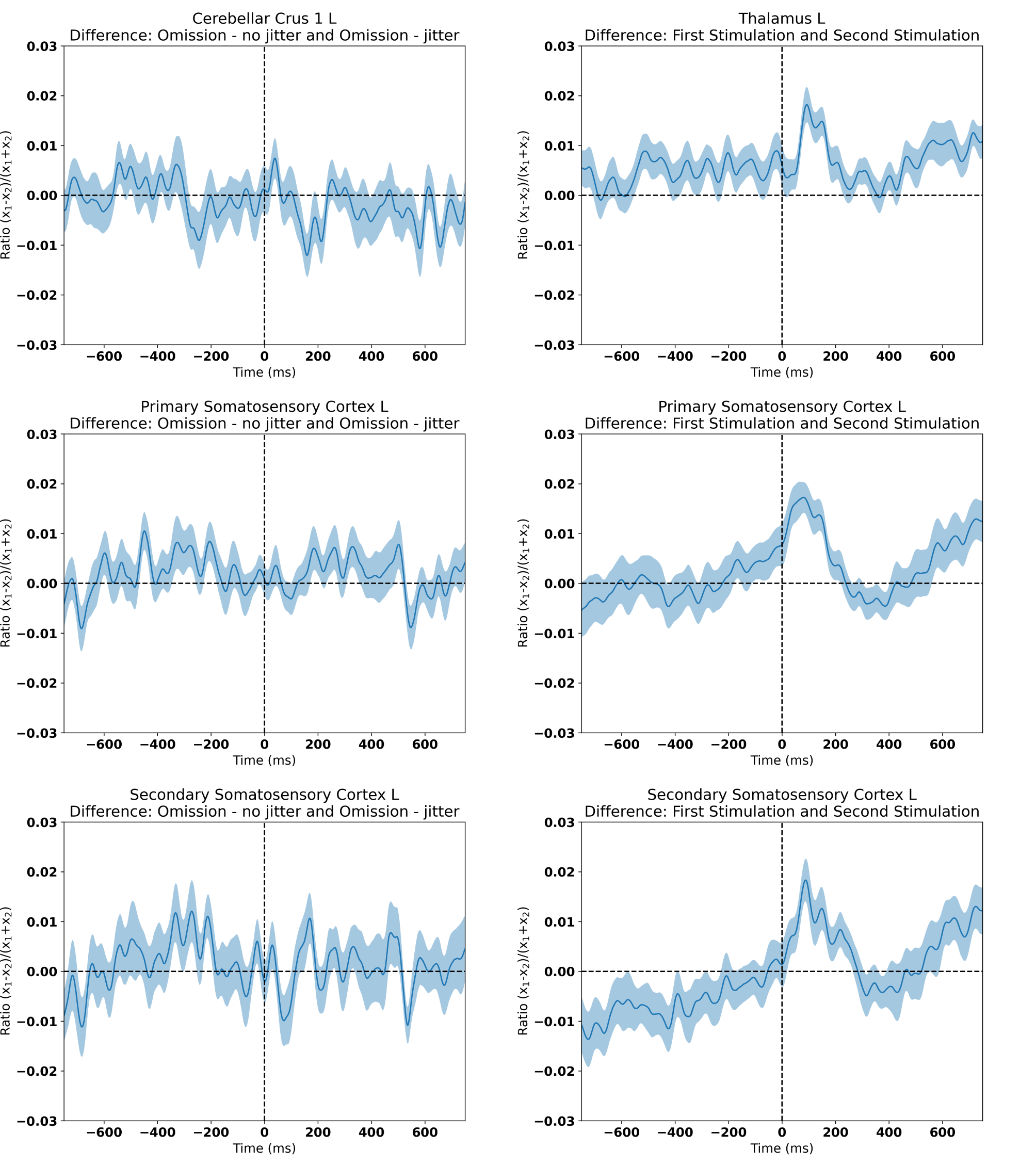

Supplement: Figure 4-1 — Beta band (14-30 Hz) ratios for omissions and stimulations respectively: The blue lines indicate the ratio (x1-x2)/(x1 + x2) for omissions (left column), no jitter and jitter, and stimulations (right column), first and second. The shaded areas indicate the standard error of the mean. Download Figure 4-1, TIF file. [file eneuro-11-ENEURO.0070-24.2024-s005.tif]
